# Supplementary material for: Enhancing Chemotherapeutic Efficacy in Lung Cancer Cells Through Synergistic Targeting of the PI3K/AKT Pathway with Small Molecule Inhibitors
Source: Int J Mol Sci. 2025 Aug 28;26(17):8378. doi: 10.3390/ijms26178378 (PMC12428413; doi:10.3390/ijms26178378)
Supplement: Supplementary file 1 [file ijms-26-08378-s001.zip › Supplementary Files for Revision.pptx]

## Slide 1
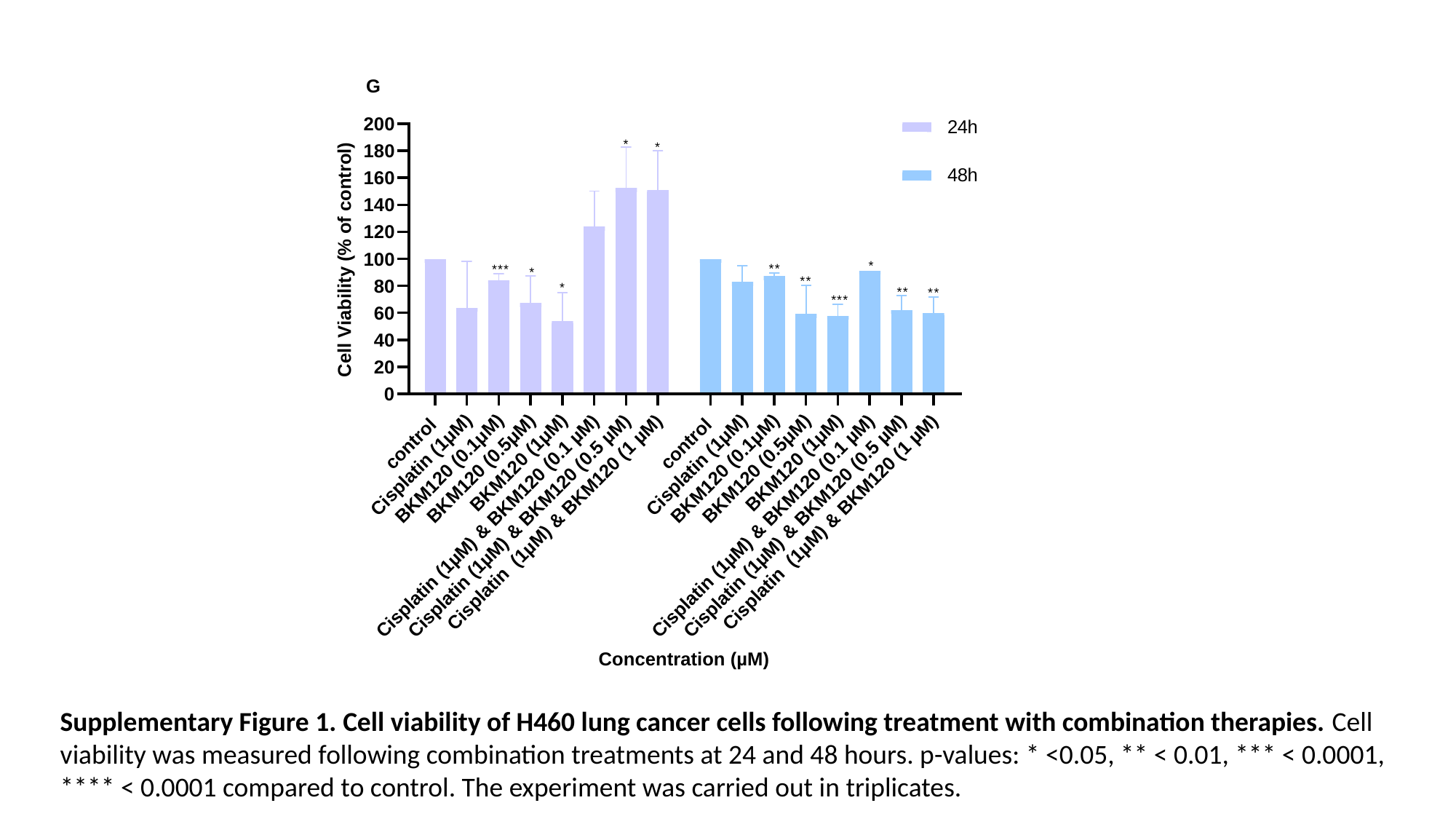

Supplementary Figure 1. Cell viability of H460 lung cancer cells following treatment with combination therapies. Cell viability was measured following combination treatments at 24 and 48 hours. p-values: * <0.05, ** < 0.01, *** < 0.0001, **** < 0.0001 compared to control. The experiment was carried out in triplicates.

## Slide 2
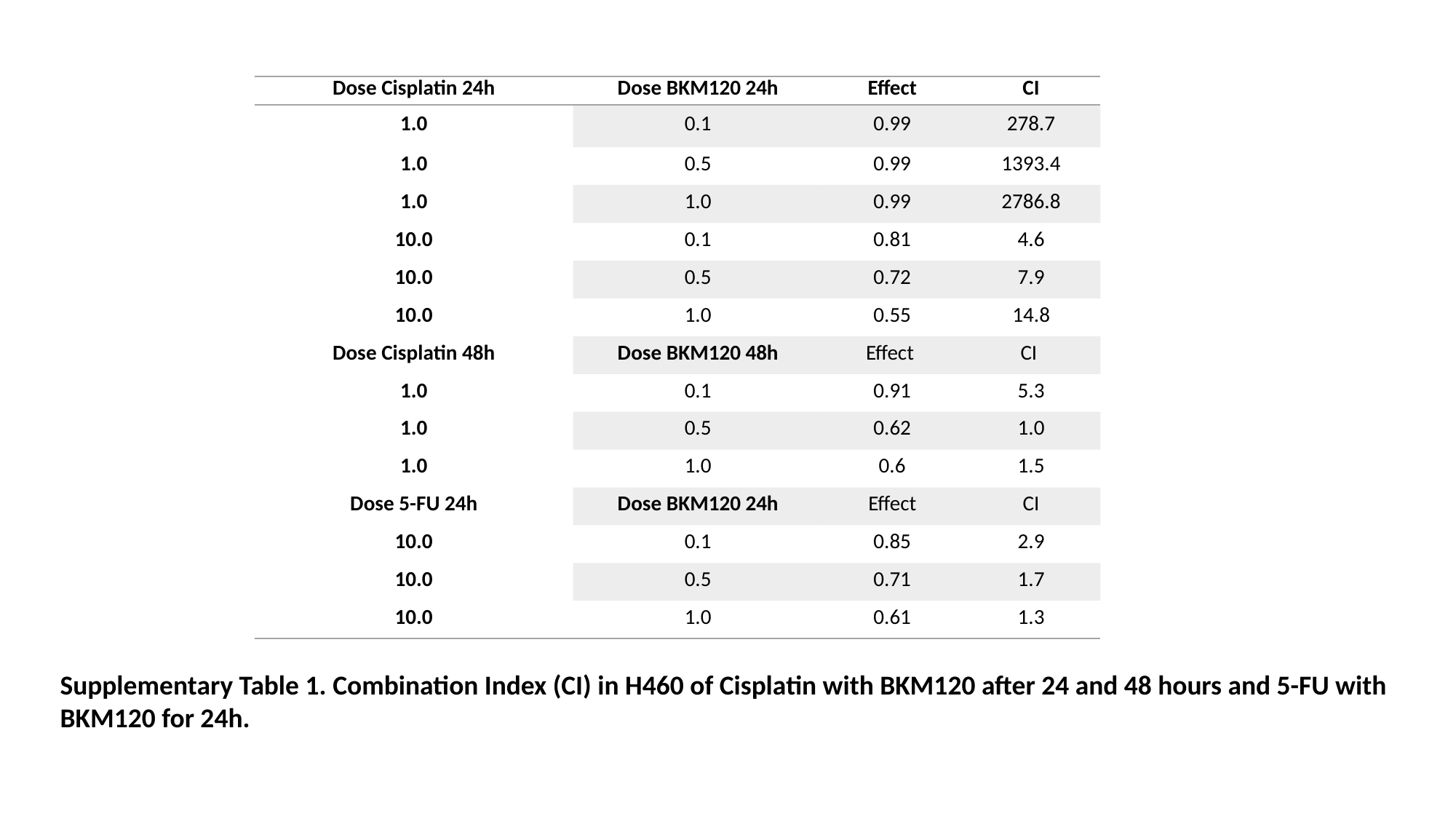

| Dose Cisplatin 24h | Dose BKM120 24h | Effect | CI |
| --- | --- | --- | --- |
| 1.0 | 0.1 | 0.99 | 278.7 |
| 1.0 | 0.5 | 0.99 | 1393.4 |
| 1.0 | 1.0 | 0.99 | 2786.8 |
| 10.0 | 0.1 | 0.81 | 4.6 |
| 10.0 | 0.5 | 0.72 | 7.9 |
| 10.0 | 1.0 | 0.55 | 14.8 |
| Dose Cisplatin 48h | Dose BKM120 48h | Effect | CI |
| 1.0 | 0.1 | 0.91 | 5.3 |
| 1.0 | 0.5 | 0.62 | 1.0 |
| 1.0 | 1.0 | 0.6 | 1.5 |
| Dose 5-FU 24h | Dose BKM120 24h | Effect | CI |
| 10.0 | 0.1 | 0.85 | 2.9 |
| 10.0 | 0.5 | 0.71 | 1.7 |
| 10.0 | 1.0 | 0.61 | 1.3 |
Supplementary Table 1. Combination Index (CI) in H460 of Cisplatin with BKM120 after 24 and 48 hours and 5-FU with BKM120 for 24h.

## Slide 3
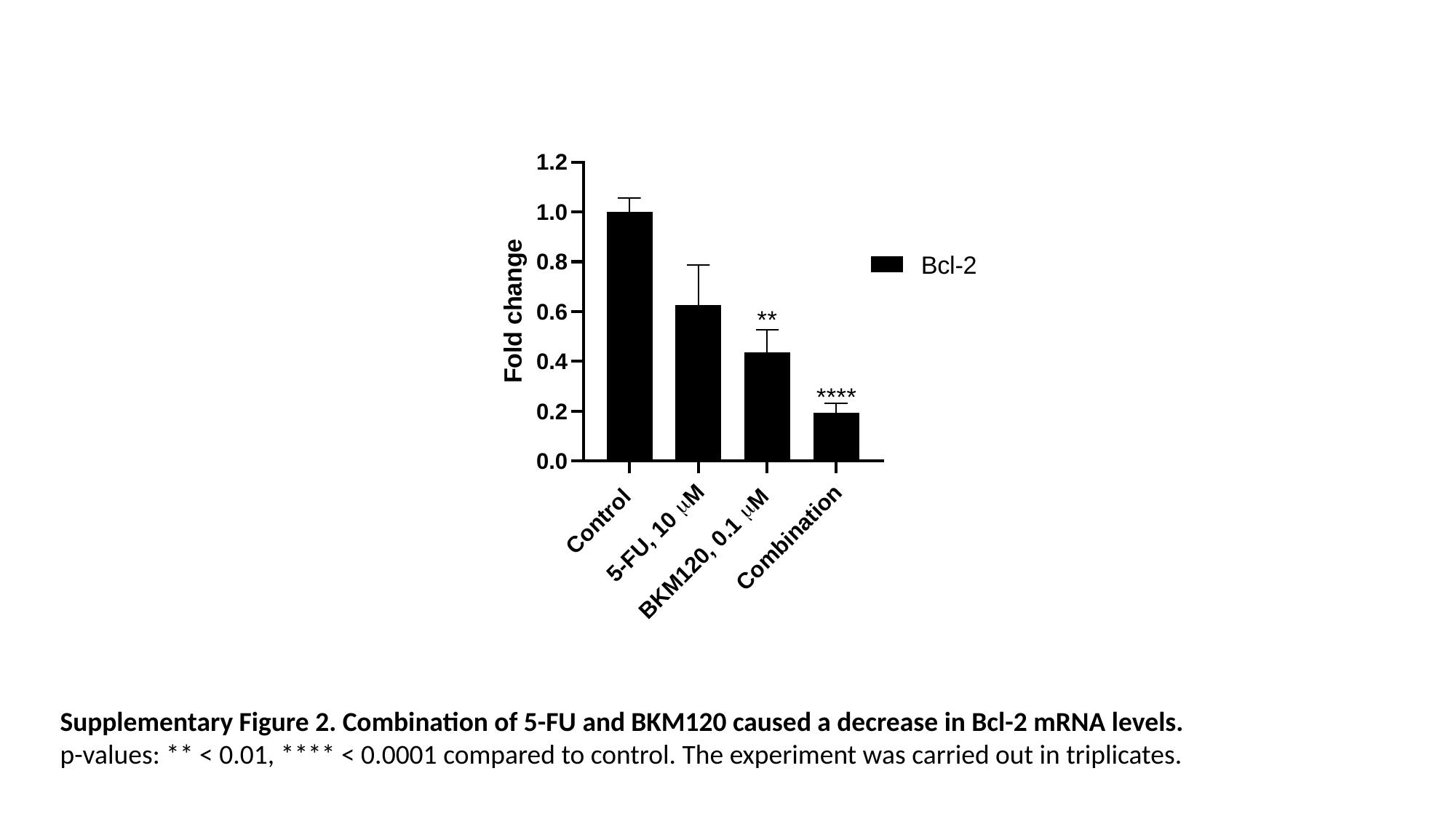

Supplementary Figure 2. Combination of 5-FU and BKM120 caused a decrease in Bcl-2 mRNA levels.
p-values: ** < 0.01, **** < 0.0001 compared to control. The experiment was carried out in triplicates.
